# Observations on Pertussis

**Published:** 1818-07

**Authors:** Nicholas Littleton


*For the London Medical and Physical Journal.*

### *Observations on Pertussis;* by Nicholas Littleton, Esq.

As No. 231 of your Journal contains Observations on Hooping-cough, perhaps a few remarks on that disease, in an early number, may not be thought undeserving of insertion.

Before proceeding to give an account of any epidemic, it seems particularly necessary to know something of the situation of the country in which such epidemic occurred, I shall therefore give a short account of Padstow. It is a sea-port on the Bristol Channel, situated in a valley sheltered by hills, about a mile and a half from the shore. Judging by my feelings, it is considerably warmer here, even in winter, than in London; frost is rarely of long continuance, and snow generally thaws within a few days after its fall. In summer, also, the heat is without doubt less scorching than in inland situations, since the constant fluctuation of the sea must have a great effect in equalising the temperature of the atmosphere. It is not to be expected that such a situation should be unhealthy, and I think it must be proved otherwise, when I mention, that in Padstow, which contains only seventeen hundred inhabitants, there are forty-five at and beyond the advanced age of seventy-five. The average number of deaths, annually, may be rather above twenty; in 1817, however, only eleven died,—and, before the peace, the marriages occasionally exceeded the deaths. The annual number of births may be between fifty and sixty. The prevailing diseases are chiefly, if not always, inflammatory, which diseases have generally attacked the respiratory organs. In the excessively hot weather of 1808, croup was epidemic, —twenty to thirty children, under four to six years of age, were attacked; since, cases of croup have occasionally occurred, but I have not known of a single case for the last three years. In 1809, peripneumonia was very general: it began about the latter end of February, first among the aged, (to whom it commonly proved fatal,) and continued till the latter end of May. During this period, between ten and twenty died of it. It is remarked here, that there are more deaths, especially among the aged, from December to May, the cold changeable weather about this time of the year causing pulmonary inflammations.

I cannot thus take a review of prevailing diseases without adverting to that most fatal one to children—variola, now disarmed of all danger by the preventive arm of Jenner. Custom has, from the remotest ages, conferred the highest honours on devoted characters—on Alexanders and Cæsars. The Old Testament savours of the same: "Saul has slain his thousands! and David his ten thousands!" but of Jenner it may hereafter be said, he has saved his millions! Customs arise, and are propped by the feelings, which are the inconsiderate impulses of the moment; these impulses, repeated from habit, become so strengthened as to form an almost irresistible law of human nature, superior even to reason itself. But can honours conferred from such sources give the happy state of mind that is connected with truly rational pursuits, which, when so successful, open into such a paradise of pleasing sympathy, extensive as is human existence? By the introduction of vaccination, there has been but one death from variola for twenty years, during which time it has been in Padstow but once, the sources by which it was more frequently introduced being cut off by vaccination. Happy would it be were this most useful preventive more generally received as prevention of the disease; for, a few years, conjoined with quarantine regulations, would remove all chance of contagion, whence vaccination also might become unnecessary. The child which died had variola confluens by natural contagion, some being still so perverse as to reject even inoculation. Whilst variola was here, in 1813, ten or twelve of those who had been vaccinated had an anomalous eruption which appeared variolous, but was of shorter duration. Five or six of these cases occurred in one family, where the children had been vaccinated at several different periods of time, and some of them have now a distinctly honey-comb eschar. Several children, however, who had had variola by inoculation, had eruptions *perfectly similar.*

Having thus digressed on variola, I return to the subject. In February and March 1816, there prevailed very generally among young children an epidemic, which shewed itself by feverishness, with cough, and very quick or rather panting respiration, and quick pulse, which symptoms amended in three or four days; and only one child, which, by an imperfection of the pulmonary functions, was blue from its birth, died.

In July 1817, a person came to visit his friends from Newquay, a village twelve miles distant. He had then the hooping-cough. He remained here only a day or two: he again visited Padstow for a short time in September, still having the hooping-cough; since which time he has lived in Wales. About December, a child in the house he visited first had a cough, and first began to hoop about Christmas; it then began to spread to the neighbouring children, proving it to be pertussis; the contagion of which had been dormant between two and three months, which may be a fact not often admitting of such clear proof, and may therefore deserve particular notice. It continued to spread; and all had the cough in a mild way, only vomiting with it, and bleeding at the nose, until about the middle of February, when one or two children that had the cough in a very trifling manner, not even hooping, became affected similarly to those who had the epidemic of 1816,—having fever, panting respiration, very quick pulse, and the cough lessened. I saw about fifteen thus affected; five of whom died. The death of one of these must not be attributed entirely to the disease, as it had been badly nourished from its birth, and was not likely to have lived otherwise.

*Case* 1.—A delicate boy, æt. four years: considered it as an attack of epidemic similar to 1816, therefore thought it might have a similar termination; but, as it continued equally bad on the fourth day, I recommended bleeding, which was not complied with. This child had never hooped; and now the cough was less violent, and only hecking.

Next day, still the same; much anxiety, quick breathing, accompanied with a moan at each inspiration. On enquiring, I learnt that the child had occasionally complained of pain in his belly; and, on asking him to put his hand to the part, he applied it to his chest. Children call all pains within, pains of the belly, or pains of the stomach, their vocabulary being as yet very limited. I now was allowed to bleed; but, failing at the first touch of the lancet, the child cried, and the mother would suffer no further trial, to which I acceded on her agreeing to allow of the application of leeches. These, however, were not applied, because the child cried, and the mother was unwilling to have it disturbed. I now despaired of the case. It continued to breathe with the same quick moaning inspiration for about two days, the pulse gradually becoming weaker, when it died.

*Case* 2.—Girl, æt. five; had had cough four or five weeks, and hooped loudly: had similar attack of fever, quick pulse, and quick breathing, but less so than in case 1. The cough continued unabated. Bled to seven or eight ounces on the third day; afterwards the cough did not seem so often, although equally violent. The breathing improved. In seven or eight days it lost the fever, and had only the cough.

*Case* 3.—Boy, æt. 22 months; had had the cough for a few weeks; now had fever, &c. Cough, much lessened. Applied nine leeches on the second day to his chest: bled copiously, but did not seem to relieve. Next day, the child had not its usual transparency of skin, from a slight degree of lividity; breathed quick as before, pulse strong and quick. The morning following, the child was convulsed: I saw it soon after, when it took no notice of things around it: countenance more livid; pulse not so strong; continued in a state of indifference to all things for about two days longer, and died.

*Case* 4.—Boy, æt. three years; treated with emetics, purges, blisters, and an irritating ointment of the tartarised antimony, ʒſs. to ʒi. to ℥i. of simple ointment, which was applied all over the chest. I saw the child on the morning of the sixth day of its illness; it was cross, and would not allow me to touch its wrist: breathing quick at times, but sometimes less quick in the evening. It died of convulsion.

*Case* 5.—Girl, æt. two years and a half, sister to Case 1; had had the cough, with trifling hooping, for two or three weeks, and had always lived in an upstair room, and been taken the greatest care of, never being exposed to cold, &c. Nevertheless, it was attacked as the others: treated as the last. When thus ill, the cough was much lessened, so that it did not open its mouth whilst coughing. As it got better the cough increased. It recovered slowly.

*Case* 6.—Girl, æt. three years; had cough, but it had not been exposed to hooping-cough: breathing eighty times in a minute: pulse 160: treated without bleeding: recovered slowly; and, after some time, the cough became more decided, even to hooping. During its illness, many lumbrici were voided. It recovered slowly. The children of a neighbour, with whom the child had been, began also to hoop; and, on enquiring, it was found that these children had been exposed to the contagion. One of these children, æt. about twelve months, had also the affection of its breathing, but recovered.

*Case* 7.—Æt. one month; had the affection of its breathing a week or two after the attack of the cough. One evening I was called, because the child was convulsed. The convulsion had ceased; but the child had frequent suspensions of respiration for nearly half a minute, which gradually became less. It lay motionless. Gave antim. tart. in solution, till it caused vomiting; for which effect, four or more grains were required, as it is often the case that children require large doses of emetic substances. I have several times known a scruple of ipecacuanha given to infants without causing vomiting. The irritating ointment was applied; and, by occasional emetics and purges, the child got better, and, as it recovered, the cough increased.

*Case* 8.—Boy, æt. eighteen months; had cough several weeks; when it was attacked as the others. Saw it on the fourth day of its illness: breathing one hundred times in a minute, with elevation of the alæ nasi at each inspiration; pulse 180; took no notice of surrounding objects; countenance as in Case 3. Gave antim. tart. till it caused vomiting. It died in the evening.

The symptoms threatening fatal termination were, anxious leaden or livid appearance of the countenance, very quick and laborious breathing, and indifference to surrounding objects. The favourable symptoms,—return or increase of the coughing, and diminished quickness of the breathing and pulse, &c.

In pertussis,—or even with this superadded affection of the mucous membrane of the lungs, which superadded affection some may consider only as a more severe form of pertussis. This severe form, however, seems less dangerous in such as have the trachea and bronchial tubes of a larger size, and are also above the ages liable to convulsion. I once knew a man, æt. 80, who had pertussis, and very mildly. From the nature of the affection, there can be no doubt of the propriety of bleeding, but emetics should not be disused; and it was remarked, that those who had much cuticular inflammation, induced by the ointment, did well.

Before and during the time that this affection prevailed, three or four cases of peripneumonia notha, or inflammation of the mucous membrane of the lungs, occurred among elderly people, one of whom, a female, rather infirm, died.

About the time that pertussis was at its height, several persons, who considered themselves passed it, were attacked with severe cough, strangling, and hooping. One, a female, æt. 30, said she had had pertussis only two years ago, at a time that it prevailed in the neighbourhood where she then lived. She was then affected severely with strangling and hooping, as she was also at this time. Now the cough was so severe, that it caused loss of vision for five or more minutes together after each severe attack of coughing. She had much head-ach, the eyelids were black, and the white of the eyes quite suffused with redness from extravasated blood.

I have chiefly intended this letter for the recording of two facts with respect to the nature of this contagion. One, the length of time it may remain dormant, which I have already mentioned, and the other, the probable necessity of almost immediate contact for its propagation. In two or three families, the children have been preserved free from the complaint by attending to this. The contagion, unlike that of variola, or rubeola, seems propagated only at small distances,—as by playing together, being in the same room, &c.; but it does not seem to spread to such distances as across a street, or to adjoining houses. Hence, parents may preserve their children free from the disease, until they have acquired greater ages, or until the season of the year is more favourable.

In the cases mentioned in the Collectanea, it is remarked, "that the breathing was short and quick, or hurried and laborious, whilst awake, but perfectly easy during sleep." This I never observed, although I saw several of them sleeping. On the contrary, the breathing was but little slower; and, in Case 6, I once counted the respirations by my watch whilst the child was sleeping.

In health, I have observed the difference in the breathing of those who were sleeping, and of those who were awake. Van Swieten, whose commentaries contain much truth, but blended with error, and whose work is chiefly to be valued as a compilation of the observations of numerous authors down to his time, in aphorism 739, has these words:— "*tria in respiratione considerantur, inspiratio, expiratio, et*

*tempus medium inter inspirationem et expirationem.*" Now, any one may observe, who attends for an instant, that the *tempus medium* (the cessation from breathing) is not after inspiration before expiration, but after expiration before the next inspiration. In health, whilst the body is perfectly at rest, inspiration is performed in a longer time than expiration, and, after each expiration, there is a short period of cessation from breathing. The duration of this period is lessened by exercise, external heat, and the exhilarating passions; and inspiration may be made to succeed instantly to expiration: the inspiration, by the same causes, becomes lessened in duration, and may thus be rendered as short as expiration. During sleep, expiration is equal to, or rather of longer duration than, inspiration. This equalization of expiration to inspiration does not arise from a quicker performance of inspiration, as after exercise, but from a slower action of the expiratory muscles. There is now no *tempus medium*, that period being taken up by expiration. I remarked, several years ago, during the insensibility that immediately succeeded a blow on the head, that inspiration was performed quick, even spasmodically, and expiration was extremely slow, even five to ten times more lasting than inspiration; and I have since remarked the same, but not to such an extent, in several cases where the vital energy was very much reduced.

There are some remarks made on the pulse, that were, perhaps, considered somewhat diagnostic,—as the grating sensation. In reading medical writings, I have been often puzzled by distinctions of pulses,—distinctions which are already beyond numbering, and still increasing. I shall here set down a few of the many that may be met with. 1. As to pulses, in relation to time; synchronous, isochronous, quick, frequent, innumerable, continuous, slow, rare, slow and crowded, intermittent, internident. 2. As to increased momentum; resisting, tense, firm, strong, full, large, open, vehement, surging, free, expansive, throbbing, bounding, thrilling. β. With hardness; resisting, tense, firm, incompressible, round, hard, sharp, wiry, chordy, vibrating, serratile, (that is said, to hack like a saw,) jerking.* 3. As to decreased momentum; imperceptible, distinct, weak, feeble, small, compressible, depressed, oppressed, soft, languid, weak and concentrated. 4. As to time and

---

* Mr. John Hunter, in the Treatise on Inflammation, says, every one cannot feel the jerking in the inflammatory pulse. Might not this have arisen from the different manner of applying the fingers; force; regular, irregular, unequal, unsteady, tremulous, undulating, vacillating, interrupted, vermicular, formicant, fluttering, redoubling,* double-striking, &c. Many of which epithets are ill applied, and some, I need not say, are impossible, as applied to pulses.

Rules for judging by pulses can only be formed from close attention. Such rules must be so depending on degree and comparison, that one could hardly lay them before the public with such confidence as one could observations, which are so plain as cannot fail to be understood by all men, whether medical or non-medical.

Now, the pulse is said to be attended with a grating sensation—grating, as I understand the word, may be either heard or felt, but is never caused but by the attrition of rough, and consequently hard, bodies; therefore it can never be felt in pulses. I can, however, guess what the author's feelings were, from what I have felt myself. Sometimes, in arterial pulsations, a sort of thrilling can be felt during the diastole of the artery: this thrilling arises merely from the manner in which you apply the fingers in order to feel the pulse. If any one should question my meaning, by the word thrilling, I would refer him to his own sensations. This thrilling can always be felt in the artery at the groin, in thin persons, by the following method: place two or three fingers on the artery, so that each finger may feel its pulsation; then press that finger which is on that part of the artery nearest to the heart so firmly as to interrupt, in some degree, the flow of blood through it, when the thrilling will immediately be felt by the fingers placed lower, and which are not compressing the artery so firmly. The cause of this thrilling, and why it is not felt in all arteries, and only in some during certain states of the body, are all easily explicable; but, as I think I have given sufficient reasons for disbelieving this grating sensation, I shall bid adieu for the present.

*Padstow; May* 26, 1818.

---

* I am not aware that it is irregular to ask questions, as was done in No. 222 of your Journal, on the redoubling pulse. As there has been no answer as yet, I should feel obliged to the editors, or any other gentlemen, for an account of such pulse, and further ask, whether it may not be similar to the double-striking pulse of old authors,—or to the *pulsus dicrotus*, or rebounding pulse, of Solano de Luque.

